# Convolutional Neural Networks enable efficient, accurate and fine-grained segmentation of plant species and communities from high-resolution UAV imagery

**DOI:** 10.1038/s41598-019-53797-9

**Published:** 2019-11-27

**Authors:** Teja Kattenborn, Jana Eichel, Fabian Ewald Fassnacht

**Affiliations:** Institute of Geography and Geoecology (IFGG), Karlsruhe Institute of Technology (KIT), Kaiserstr. 12, 76131 Karlsruhe, Germany

**Keywords:** Biodiversity, Community ecology, Invasive species, Forestry

## Abstract

Recent technological advances in remote sensing sensors and platforms, such as high-resolution satellite imagers or unmanned aerial vehicles (UAV), facilitate the availability of fine-grained earth observation data. Such data reveal vegetation canopies in high spatial detail. Efficient methods are needed to fully harness this unpreceded source of information for vegetation mapping. Deep learning algorithms such as Convolutional Neural Networks (CNN) are currently paving new avenues in the field of image analysis and computer vision. Using multiple datasets, we test a CNN-based segmentation approach (U-net) in combination with training data directly derived from visual interpretation of UAV-based high-resolution RGB imagery for fine-grained mapping of vegetation species and communities. We demonstrate that this approach indeed accurately segments and maps vegetation species and communities (at least 84% accuracy). The fact that we only used RGB imagery suggests that plant identification at very high spatial resolutions is facilitated through spatial patterns rather than spectral information. Accordingly, the presented approach is compatible with low-cost UAV systems that are easy to operate and thus applicable to a wide range of users.

## Introduction

Accurate information on the spatial distribution of plant species and communities is fundamental for various fields of application, including research, nature conservation management, forestry, agriculture, or ecosystem service assessments. In this regard, remote sensing technologies have evolved as a promising tool and continue to develop at unpreceded pace^1–4^. Due to novel sensors and platforms, such as very high-resolution satellite missions or Unmanned Aerial Vehicles (UAV), there is a growing availability of optical earth observation data revealing both high spatial and temporal detail on vegetation patterns^5^. Using photogrammetric techniques, this optical feature space can further be extended with information on the 3D structure of vegetation canopies^6–8^. Efficient methods are needed to fully harness this unpreceded source of information for vegetation mapping.

In this regard, deep learning, also known as self-learning artificial intelligence approaches, are paving new avenues for data analysis and computer vision^9^. In the field of remote sensing, so-called Convolutional Neural Networks (CNN) are currently revolutionizing possibilities for object detection and pattern recognition^10,11^. In contrast to common pixel-based methods, CNN allow for an efficient analysis of image textures, i.e., the contextual signal of multiple neighbouring pixels. The self-learning capabilities of CNN enable an efficient analysis of such textures, which in turn can reveal the decisive leaf and canopy traits required for identifying vegetation communities or species^12,13^. Thus, it is expected that in tandem with advances in high-resolution sensor technology, CNN will revolutionize our capabilities to map vegetation patterns^14^.

CNN have been initially designed for image categorization tasks^15^. Examples from the field of vegetation science are apps like *Flora Incognita* or *Pl@ntNet*, which assign a species name to plant photographs^12,16,17^. Such CNN autonomously extract the contextual features of an image dataset and learn which of these features (e.g,. leaf forms or traits of the flowers) are relevant for assigning the observations to the specified categories. Given the myriad of ways and scales to characterize spatial context^18^, the self-learning capabilities of CNN are a great advantage in terms of computational efficiency and automatization since a feature design process is not required^10^. A key constituent of common CNN architectures for identifying such features are multiple and subsequent pooling operations that aggregate the feature maps derived from convolutions to a coarser spatial scale, and thereby increase the robustness and efficiency of the network. The last layer of the network will simply contain the (aggregated) information whether a feature that is indicative for the target class was visible anywhere in the image or not.

**Figure 1:**
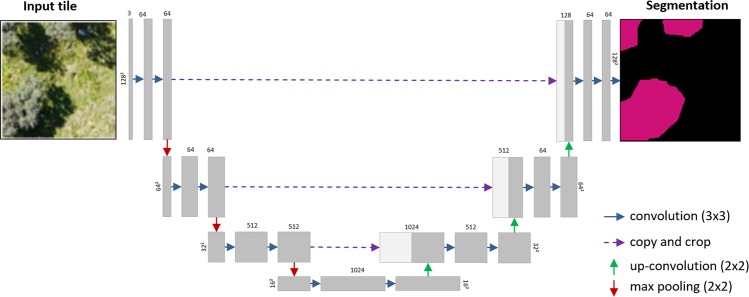
Scheme showing the CNN-based segmentation implemented using the U-net architecture. The input tile (left), an extract of 128 × 128 pixels, is input for multiple convolutions (encoding layers), which extract decisive spatial patterns at the respective scale (grey). Subsequently, the feature maps are resampled (max pooling) to a coarser resolution before the next convolution process is being applied (etc.). Eventually, the spatial features learned at the different spatial scales are combined through a series of decoding layers (up-convolutions) to segment the spatial extent of the target class (right, pink).

Yet, the above-described mode of CNN-based categorization of entire images is not appropriate for remote sensing-based vegetation mapping, where the goal is to provide spatially-continuous, fine-grained classifications within the extent of an image (e.g., an airborne mosaic). Here, the question is not whether the target class is present, but where it is present. Ideally, such classifications are performed at the original resolution of the remote sensing imagery to preserve spatial detail. In this regard, a powerful CNN-based approach for fine-grain image classification is given by fully convolutional networks, as these remember and reconstruct the position of the contextual features through an encoder/decoder mechanism (Fig. 1). Fully convolutional networks thus enable the extraction of contextual features within a wide receptive field (here an extract of an orthoimage), while preserving the spatial origin of these features to produce a fine-grained and spatially explicit segmentation of the object^10,19^.

One of the most successful fully convolutional network architectures is the *U-net*^19^ (Fig. 1), as has been demonstrated in several contests, such as the *Inria Aerial Image Labelling* (*IAIL*) Benchmark^10,11^. A few pioneering studies have already indicated the potential of the *U-net* architecture for vegetation mapping^13,14^. Yet, the application of the *U-net* and other fully convolutional network architectures remains sparse, and the potential is not fully explored. This may partly be explained by a general bottleneck of CNN training: the need for ample reference observations for identifying and learning the decisive image features^20,21^. In remote sensing of vegetation, reference observations are commonly acquired in the field, involving high logistic efforts, inaccuracies due to geolocation errors and sampling and observation bias^22–24^. A promising alternative for an efficient reference data collection in the field is given if the spatial resolution of the remote sensing imagery enables the visual identification, and thus, delineation of training data directly in the images^6,13,25^.

Accordingly, we test if a CNN segmentation approach (*U-net*) and training data derived from visual interpretation allow for robust and fine-grained mapping of vegetation species and communities in high-resolution UAV data. For this task, we only consider high-resolution UAV data derived from off-the-shelf RGB sensors, including 3D information derived with photogrammetry. The segmentation accuracy is tested for three growth forms, i.e., i) herbaceous plant communities along a successional gradient, ii) a shrub species (*Ulex europaeus*) and iii) a tree species (*Pinus radiata*).

## Results

The accuracy of the CNN-based segmentation process was assessed using independent validation data derived from visual interpretation, and is presented in Table 1. Overall, we found the segmentation to be very accurate (>84%) for all growth forms and target classes considered, i.e., mapping of herbaceous vegetation communities, the shrub species *Ulex europaeus* and the tree species *Pinus radiata*. Systematic segmentation errors were not observed, as indicated by the low bias values, which were calculated by averaging the residuals of the prediction derived from the independent validation data.

**Table 1:** Predictive performance of segmenting the target classes using the U-net architecture.

Target class	Accuracy [%]	Bias
*Pinus radiata*	87	0.0024
*Ulex europaeus*	84	−0.0662
Pioneer community 2	90	0.0529
Intermediate successional community 2	89	0.0401

**Figure 2:**
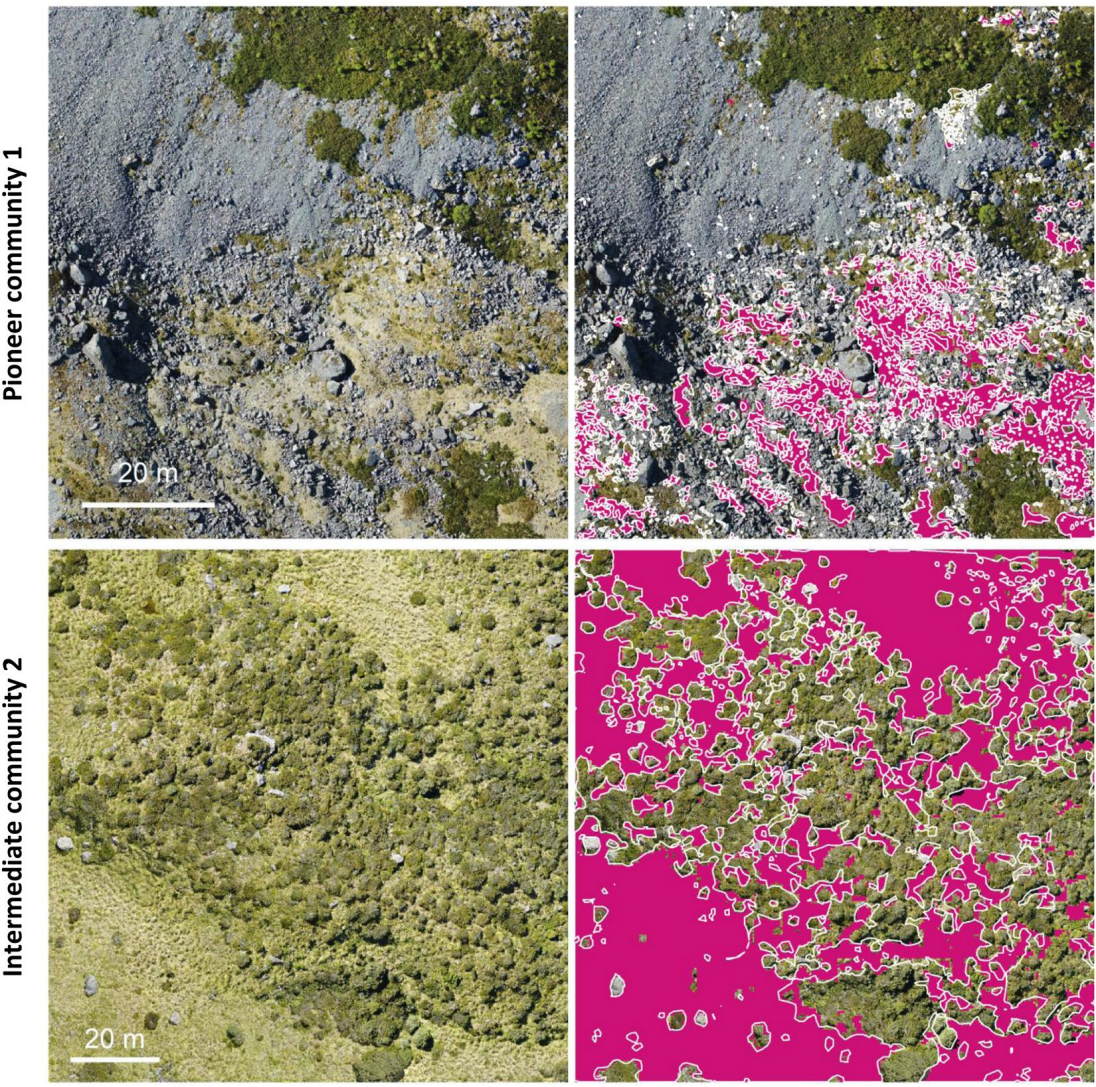
Mapping herbaceous vegetation communities along a successional gradient, i.e., a pioneer vegetation community (top) and an intermediate vegetation community (bottom) in the Mueller glacier foreland. Left: The input RGB imagery used for the CNN-based segmentation. Right: The reference data (white polygons) and the segmentation results (purple). Centre coordinates top: 1366085, 5156801; bottom: 1365921, 5157281, EPSG: 2193.

**Figure 3:**
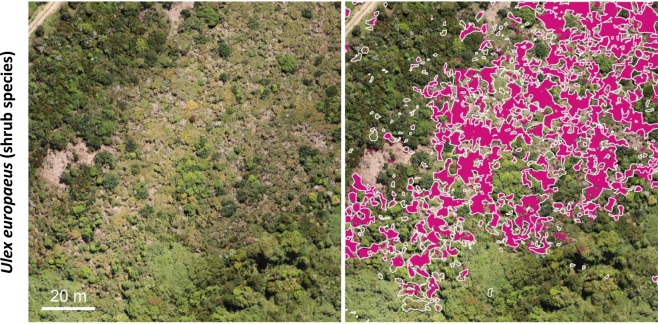
Mapping the shrub species *Ulex europaeus* in central Chile. Left: The input RGB imagery used for the CNN-based segmentation. Right: The reference data (white polygons) and the segmentation results (purple). Centre coordinates: 587385.4, 5363396, EPSG 32718.

**Figure 4:**
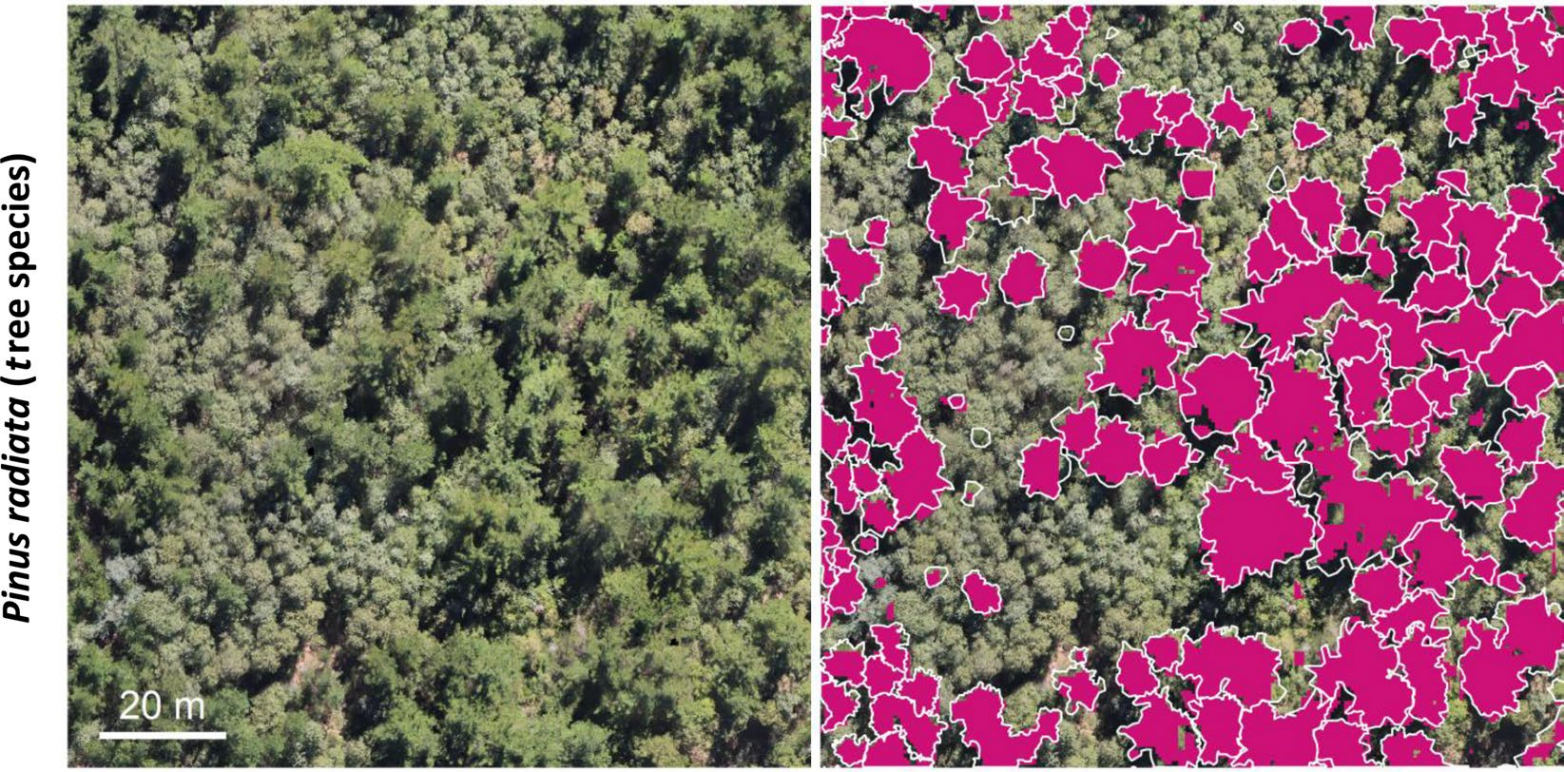
Mapping of the tree species *Pinus radiata* in central Chile. Left: The input RGB imagery used for the CNN-based segmentation. Right: The reference data (white polygons) and the segmentation results (purple) Centre coordinates: 208876, 6082517, EPSG 32719.

Maps of the predictions, together with the visual delineated reference data, are shown in Figs. 2–4 and reveal a very high correspondence. Overall, the predicted patterns do include few false negatives and false positives, i.e. missing segmentation within the reference polygons or segmentation outside the reference polygons. False positives usually feature a small size. Likewise, false negatives are mostly found for small-sized canopies of the target class. Almost no false positives or negatives were found for medium or large canopies of the target class. For *Pinus radiata*, we observed a few cases where occurrences of very dark cast shadows, resulting from large crowns and dense canopies, show an increased chance of false negatives. The results for *Ulex europaeus (*Fig. 3) as well as the Pioneer community (Fig. 2) appeared to be noisier, especially in case of gradual transitions and in the presence of sparse canopies.

## Discussion

The results demonstrate that the CNN-based segmentation approach using the *U-Net* architecture accurately predicts plant species and communities in high-resolution orthoimagery. Our results are thus in line with previous studies that have demonstrated the high value of deep learning for vegetation mapping using remote sensing imagery^14,19^. Although we used only data acquired with off-the-shelf RGB sensors, the *CNN-*based segmentation procedure proved to be very accurate. The results obtained here even exceeded classification accuracies that have been derived on the same test sites using pixel-based methods (*MaxEnt)* together with comparably expensive UAV-based hyperspectral imagery^6,25^ (data only available for *Pinus radiata* and *Ulex europaeus*). The accuracy of these pixel- and hyperspectral-based classifications has not exceeded 62.5% for *Ulex europaeus* and 65.4% for *Pinus radiata*. The clear superiority of the CNN-based approach with RGB data indeed seems very plausible considering that 1) our human cognition, as well restricted to RGB information, also enables us to visually differentiate the target classes and 2) considering that CNN have been found to resemble the functioning of our visual cortex^26,27^. Our results thus show that high-resolution spectral information (multispectral or hyperspectral data) might not be an essential requirement for identifying vegetation species or communities at the spatial scales considered here. This somewhat contrasts the experience that has emerged from studies that have used comparably coarser airborne or satellite imagery with pixel-based approaches to classify plant species or communities, where a high spectral resolution is commonly expected a prerequisite for a high classification accuracy^28–30^. In fact, pixel-based classification approaches have even been found to be unfavourable at high spatial resolutions due to increasing within-class-heterogeneity^6,31–33^. Accordingly, our results indicate that with higher spatial resolution, spatial patterns resulting from traits such as leaf forms, branching patterns, and canopy shapes can be more important to differentiate certain vegetation species or communities than the overall reflectance properties of the plant canopy. This finding is also supported by previous studies that have demonstrated the potential of close-range RGB imagery (e.g., as derived from smartphones) for plant species identification^12,21^. Synergies of high spatial resolution RGB information and accurate pattern recognition by deep learning algorithms may hence develop as a key technology for operational remote sensing applications. This is particularly likely since remote sensing platforms collecting RGB data are comparatively cheap and easy to operate, and the obtained data do not require sophisticated pre-processing.

The spatial resolution of the orthoimagery available in this study ranged from 3 to 5 cm (ground sampling distance), which proved to be sufficient for an accurate segmentation of the species and communities considered here. Yet, it seems plausible that increasing the spatial resolution can further enhance accuracy as thereby more relevant contextual features can be extracted. For some species or communities, a higher spatial resolution might be even strictly necessary to reveal the decisive plant traits (e.g., leaf forms or branching characteristics), as suggested by earlier experiences from close-range remote sensing^16,21,33^.

The spatial resolution of the remote sensing imagery also determines if a classification can reveal the spatial patterns of the target class^34^. If pixel size extensively exceeds the dimensions of plant organs or canopy components, a binary segmentation into absences and presences may not be appropriate. In that case, the pixel cannot be explicitly assigned to one class. This issue may be especially critical in the presence of heterogeneous, overlapping canopies^33,35^. Likewise, the results of the current study suggested that for some areas, a classification may be limited in representing the actual vegetation patterns. This particularly applies to smooth transitions among vegetation classes as found for the herbaceous plant communities (see RGB imagery, Fig. 2) as well as for rather sparse canopies of *Ulex euopaeus* (Fig. 3). The applicability of binary segmentation can thus be improved by increasing the spatial resolution of the imagery. However, increasing the spatial resolution comes at the cost of decreased area coverage and may hence critically constrain the overall efficiency of a remote sensing application. In such a case, the mapping of continuous cover values [%] instead of discrete classes might be more appropriate. In a previous study (manuscript in preparation), it was shown that CNN also allow predictingt the cover [%] of plant species and communities for regular image tiles of the UAV orthoimagery. Yet, this regression approach is limited in terms of the spatial detail of the mapping product, as the cover values can only be well predicted for a tile size that includes sufficient contextual features. Given recent and rapid technological advances in remote sensing sensor and platform technologies, very high-resolution data will likely be easy to acquire in the near future, increasing the applicability of segmentation approaches^5^.

One drawback of these very high-resolution data is their typically limited area coverage^36^, which constrains the immediate value of resulting map products for ecology, conservation, or resource assessments. Yet, the value of such high-resolution map products can be expanded through a combination with large-scale earth observation data. Local high-resolution map products can be used to train machine learning algorithms that use spatially coarser earth observation imagery as predictors allowing to generate predictions for large-scale assessments^6,37^.

The current study highlights the potential of CNN towards applied remote sensing tasks, such as mapping invasive species and assessment of vegetation succession in a conservation area. Yet, our results also reveal limitations of the presented approach. We found that small-sized canopies were more likely to be segmented incorrectly. Furthermore, and in accordance with previous studies^25,38^, we found that the chance of false negatives increases in very dark cast shadows. This may impair the identification of small-sized individuals in canopy gaps. Both factors may constrain the accuracy of identifying small-sized individuals in multi-layered canopies, such as for mapping very early plant invasions. It is recommended to acquire imagery around solar noon to minimize the effects of cast shadows on classification tasks^25^. Cast shadows can be reduced further by acquiring data under cloud cover and diffuse light conditions, respectively. However, diffuse light conditions are also likely to reduce the decisive signal in image textures emerging from leaf, branch, and canopy traits that contribute to a separation of the target class.

Ample reference data is known to be a prerequisite for deep learning-based plant identification^12,13,21^. In this study, we trained the CNN models using reference data acquired by visual interpretation. The visual delineation was not only efficient, but also advantageous in contrast to conventional field-based ‘ground truth’ sampling as it (1) is not affected by spatial inaccuracies, (2) it is less affected by site-accessibility and sampling bias, (3) unlike plot data it is spatially explicit enabling the direct use with the orthoimagery and (4) it features a higher correspondence with the remote sensing-based predictors, facilitating statistical links in the model training^6,22–24,39,40^. Although the visually delineated canopies have been cross-checked by at least one other interpreter, inaccuracies are expected. Such inaccuracies may affect the training of the models as well as their validation. Yet, as found in a previous study^6^, it can be expected that empirical models can compensate certain degrees of erroneous reference data. It is essential to consider that the reference data acquisition using visual delineation is only applicable if the target species or community is clearly identifiable in the imagery. This will not only depend on the quality of the imagery (e.g., spatial resolution) but also the uniqueness of the morphological traits of the vegetation of interest. Yet, in any case, a CNN-based identification of plant species is only applicable if such morphological traits are present in the plant canopy.

For the present study, we tested the semantic segmentation with a CNN architecture resembling  the original U-net implementation^19^. We choose the U-net architecture since it is computationally very efficient and delivers good results with small amounts of reference data. Despite high accuracies demonstrated here, it is important to consider available options that can potentially improve the segmentation performance. This includes the use of pre-trained networks^41^, additional architecture components^42,43^, or post-processing^44,45^. Depending on the input data, other semantic segmentation algorithms than the *U-net* might be advantageous, such as *DenseNets*^46^, *LinkNet*^47^, or *RefineNet*^48^. In the context of photogrammetric data as derived from structure from motion processing pipelines, a promising avenue may be semantic segmentation algorithms that support point clouds, such as the *PointNet* algorithm^49,50^. An alternative to pure semantic segmentation is instance segmentation (e.g., *Mask_R-CNN or COB)*, which enables to delineate single entities of a class (e.g., an individual tree)^51–53^. Given this wide variety of options, careful consideration of the available data, the processing time, the desired complexity, and thus transferability should be made before choosing an appropriate CNN-based segmentation strategy and algorithm.

## Conclusion and Outlook

We demonstrated that CNN can accurately map plant species and vegetation communities from high-resolution RGB data. Our mapping results suggest that plant identification at very high spatial resolutions is facilitated through spatial patterns rather than spectral information. This is opposed to remote sensing applications at coarser spatial scales, where spectral resolution is an important criterion for plant identification. Combining high spectral and spatial resolution may even lead to superior results than achieved here. As the presented approach differentiated vegetation very accurately in RGB imagery, it is compatible with low-cost UAV systems that are easy to operate, and hence, applicable to a wide range of users. It can be assumed that CNN-based mapping of plant species and communities will pave new avenues for various vegetation-related remote sensing applications, including mapping of invasive or endangered species, habitat mapping, or resource assessments in forestry and agriculture.

## Methods

**Figure 5:**
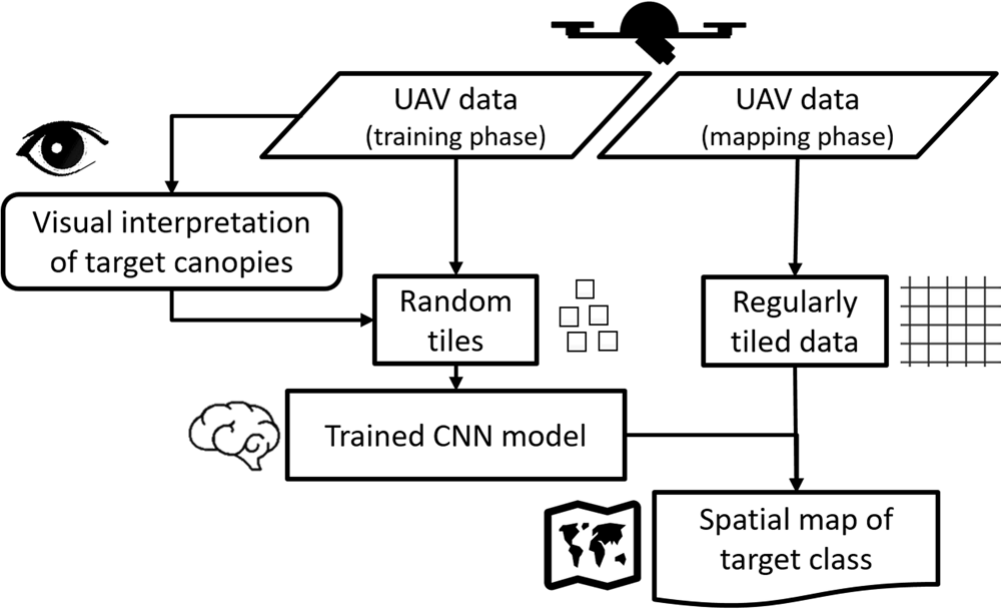
Overview of the work-flow used for CNN training and its application to UAV imagery.

The applied work-flow is centered on very high-resolution orthoimagery, and digital elevation models (DEM) derived by photogrammetric processing of UAV-based RGB aerial imagery (Fig. 5). Based on the orthoimagery, we created spatially explicit reference data for each target class by visual interpretation. We then used reference data together with the orthoimagery and DEMs to train the CNN-based segmentation of each target class (plant species or community) using the *U-net* architecture (Fig. 1). In the mapping phase, the trained CNN models are applied to the photogrammetric data using image tiles extracted by a regular grid. The resulting segmentation output is merged to produce a spatially continuous map of the target class.

### Acquisition of high-resolution imagery and visual delineation of reference data

High-resolution imagery was acquired using copter-type UAVs equipped with RGB cameras. UAVs were operated autonomously in parallel flight stripes with a minimum forward overlap of 75% and a minimum side overlap of 50%. The datasets for *Ulex europaeus* and *Pinus radiata* were acquired within the project SaMovar, which aimed to assess invasion patterns of these exotic species in Central Chile. Flights were carried out in areas with early invasions. The flight campaigns were performed using an Okto-XL (HiSystems GmbH, Germany) UAV equipped with a Canon 100D with an 18 mm lens. The flight campaigns took place in Chilean spring (March 2015) for *Pinus radiata* and summer (November 2016) for *Ulex europaeus*. The autonomous flights were performed at an average height of 150 m resulting in a spatial resolution of about 3 cm. For the present study, four flights per species were selected to test the CNN-based segmentation approach. The area covered per flight ranged from 21 to 37 hectares. For more details on the data acquisition for these two species see Kattenborn *et al*.^6^.

The data for the herbaceous plant communities were acquired in the Mueller glacier foreland, located in Mount Cook National Park (New Zealand). The Mueller glacier foreland has a size of approximately 450 ha and is shaped by a sequence of lateral and latero-frontal moraines formed between 125 y BP and 3,370 ± 290 y BP^54^. Previous studies on vegetation succession in the adjacent East Hooker Valley found distinct plant communities on differently aged terrain with pioneer and early successional stages characterized by the herb *Epilobium melanocaulon* and the moss *Racomitrium lanuginosum*, intermediate successional stages with *Festuca* and *Chionochloa* grassland and later successional shrubland with woody *Dracophyllum spp*.^55^. In order to cover the entire glacier foreland, seven individual but partly overlapping image flights were performed with a DJI Phantom 4 Pro+. The area covered in each flight ranged from 20 ha to 50 hectares. We set the operating altitude to 100 m above ground to ensure an image resolution of at least 3 cm.

For all datasets, we derived the orthoimagery using the Structure from Motion (SfM)-based photogrammetric processing chain in Agisoft Photoscan (Agisoft, Russia, vers. 1.4.2). The processing included the filtering of blurry images, image matching, and the creation of dense point clouds. The dense point cloud was then used to derive DEM on which the single image frames were projected to obtain a georectified orthomosaic. Automatic georeferencing was performed based on the GPS trajectories logged during image flights. Both the orthoimagery and the DEM were exported using the same spatial resolution (cm). Overviews of all orthoimagery are given in Supplementary Information 1.

For *Pinus radiata* and *Ulex europaeus* we acquired reference data in form of absences and presences of the respective species canopies. For the dataset on herbaceous plant communities, a prior classification into floristic classes was performed based on vegetation survey data. Plant species cover was recorded in 55 plots (2 × 2 m), which were randomly distributed in five strata representing successional stages generated from a Normalized Difference Vegetation Index product derived from a Sentinel-2 scene. The species cover data was classified into five floristically classes defined using *Isopam* (available as R-package, distance Bray-Curtis)^56^. For this study, we focused on one pioneer and one intermediate successional plant community. Results of all vegetation classes are presented in the Supplementary Information 2.

We generated the reference data for training the CNN models using GIS-based visual interpretation of the orthoimagery and the DEM. The reference data was created using polygons defining the boundaries of the target canopies. Geotagged photographs acquired during the field work were used to aid the visual interpretation. The plant species and communities of interest were usually clearly identifiable in the imagery. Only in very few areas, characterized by very dark cast shadows or blurry SfM-reconstructions, the visual interpretation was challenging. All delineated areas were cross-checked by at least one other interpreter to ensure the robustness of the reference data. For the datasets of *Ulex europaeus* and *Pinus radiata,* the target species were delineated for the entire extent of the orthoimagery. Given the large spatial extent of the Mueller glacier forefield, reference data acquisition for the herbaceous plant communities was restricted to 7 plots of 150 × 150 m (see Supplementary Information 1).

### CNN-based mapping of target classes

For the CNN-based segmentation, we implemented the *U-net* architecture^19^ (Fig. 1). The CNN was trained using tiles with a dimension of 128 times 128 pixels. 4000 tiles were randomly sampled from the orthoimages using a regular grid with a 5 m spacing to avoid overlap. The extracted tiles included the RGB bands, the DEM (explaining variables), and the delineated polygons in form of a binary mask (dependent variable). The absolute height values of the DEM were normalized to relative values (0–255) to circumvent terrain effects. 66.6% of the tiles were used for training the model, whereas 33.3% were used for validation.

The *U-net* was implemented using an R interface (version 2.2.4, R Core Team 2018) and the Keras API with the *TensorFlow* backend^57^. The models were trained on a workstation with a CUDA-compatible NVIDIA GPU (GeForce GTX 980 Ti). The *U-net* architecture featured 5 layers in which 3 × 3 convolutions were applied to derive the feature maps (Fig. 1). A max pooling operation was implemented between each convolution, reducing the spatial dimensions of the feature map by a factor of 2. The depth of the feature maps derived from the convolutions was doubled with each pooling operation. The derived feature maps, comprising the extracted spatial features at each spatial scale, are combined by upsampling operations (up-convolution) to eventually predict a segmentation output at the original dimensions of the input tiles (128 × 128 pixels). Given the binary classification problem (absence = 0, presence = 1), the final layer was activated using a sigmoid function. As optimizer, we choose the RMSprop with a learning rate of 0.0001. In view of the 2-dimensional classification task (masks vs. segmentation output), the dice-coefficient was chosen as loss function^58^. To increase the robustness and transferability of the segmentation, the tiles were subjected to a data augmentation prior to training. The data augmentation included the random shearing (0–0.2 radians), rotation (20-degree steps), shifting (0–15%), or horizontal and vertical flipping of image tiles. Besides inflating the training data quantity, this procedure inter alia allows to avoid that the CNN considers spatial features that solely depend on the data acquisition settings, e.g., the direction of the sun and shadows.

We trained the CNN models in 20 epochs with 50,000 steps and a batch size of 16. The model weights of an epoch were only considered if the accuracy surpassed the accuracy derived in the previous epoch. For testing the model accuracy of each epoch, a 20% hold-off of the training tiles was used. We assessed the accuracy of the final model using the validation data, i.e., those tiles that were not used for training the CNN model. The accuracy was quantified by calculating the number of pixels that were accurately predicted. In order to avoid bias due to class distributions, we stratified the validation data into three classes before calculating the accuracy, i.e., tiles including 0–33%, 33–66% and 66% to 100% cover of the target class.

For visual comparison of the predicted segmentation, and the visually delineated reference data, we applied the trained CNN to independent extracts of the orthoimagery (not used for training) of 120 × 120 m. Therefore, the 120 × 120 m extracts were tiled using a regular grid with each grid cell having a 128 × 128 pixel size (corresponding to the tile size used for training the CNN). We applied the CNN to each tile and merged the resulting segmentations of all tiles to a new raster layer, i.e. a segmentation map having equal dimensions as the extract of the orthoimagery (120 × 120 m, 3 cm pixel size).

## Supplementary information

Supplemental information

## Data Availability

The data that support the findings of this study are available from the corresponding author upon reasonable request.
