# Supplementary material for: Convolutional Neural Networks enable efficient, accurate and fine-grained segmentation of plant species and communities from high-resolution UAV imagery
Source: Sci Rep. 2019 Nov 27;9:17656. doi: 10.1038/s41598-019-53797-9 (PMC6881311; doi:10.1038/s41598-019-53797-9)
Supplement: Supplementary file 1 — Supplemental information [file 41598_2019_53797_MOESM1_ESM.pdf]

Supplementary information: Convolutional Neural Networks enable efficient, accurate and fine-grained segmentation of plant species and communities from high-resolution UAV imagery

Teja Kattenborn[1]*, Jana Eichel[1], Fabian Ewald Fassnacht[1]

1 Institute of Geography and Geoecology, Karlsruhe Institute of Technology (KIT), Kaiserstr. 12, 76131, Karlsruhe, Germany

Supplementary information 1: Overviews of the UAV orthoimagery for each case study.

The following figures show the orthophotos used for each of the case studies. Areas that featured artefacts of the photogrammetric processing (e.g. due to little overlap or steep viewing angles) were masked prior to analysis

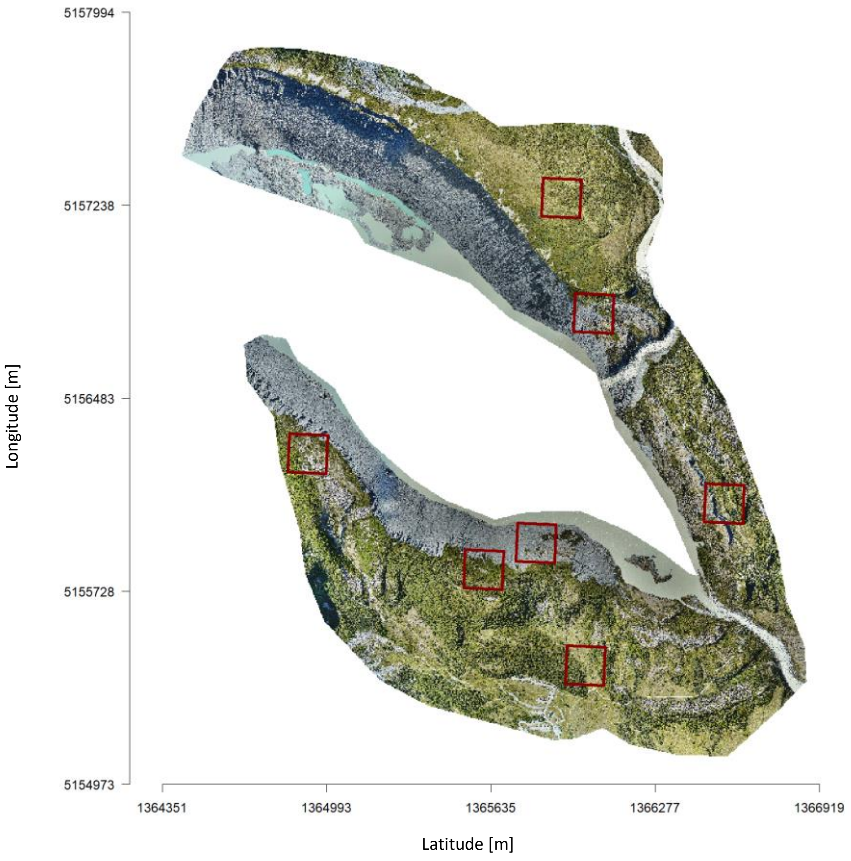

Fig S3-1: Orthoimage of the Mueller glacier forefield used for mapping successional classes (EPSG: 2193). We restricted our data analysis to the seven plots (dark red), due to the wide extent of the orthoimage.

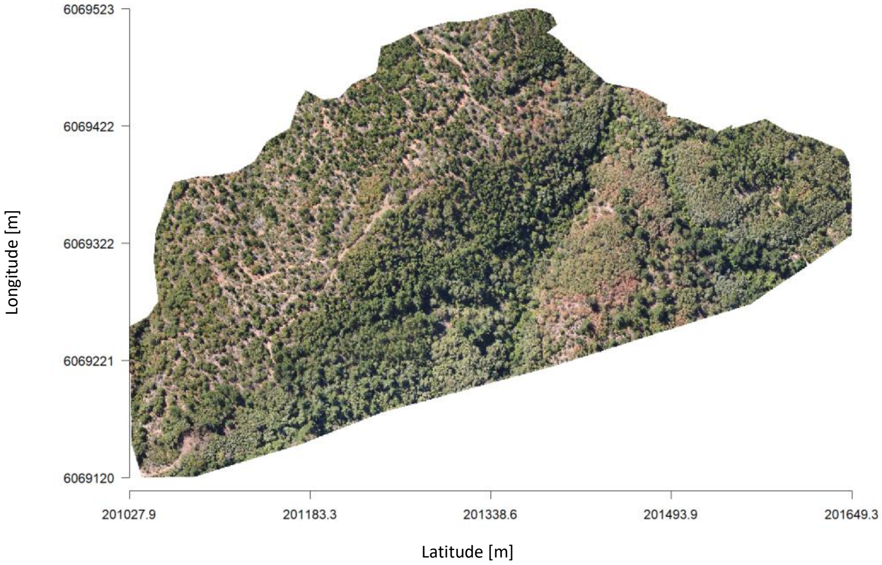

Fig S3-2: Orthoimage 1 for mapping invasions patterns of *Pinus radiata* (EPSG: 32719 XXX)

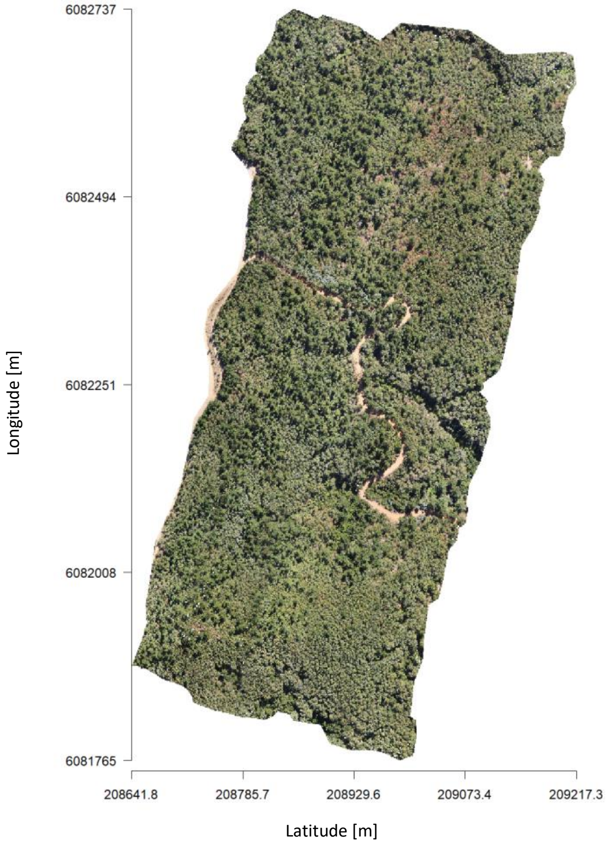

Fig S3-3: Orthoimage 2for mapping invasions patterns of *Pinus radiata* (EPSG: 32719)

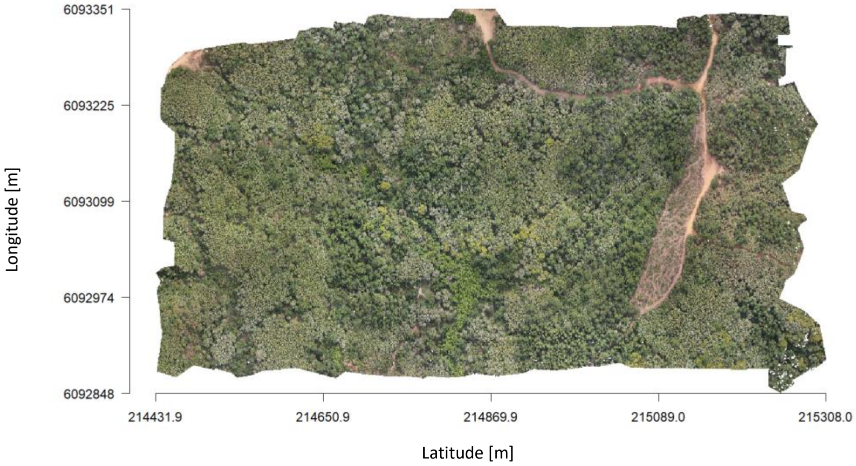

Fig S3-4: Orthoimage 3 for mapping invasions patterns of *Pinus radiata* (EPSG: 32719)

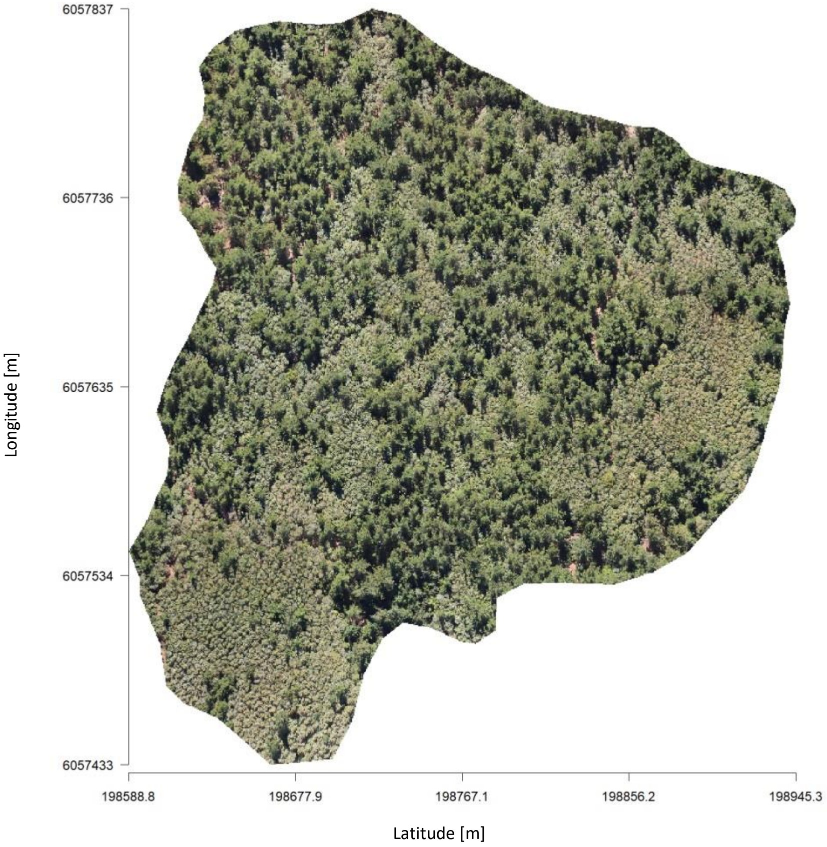

Fig S3-5: Orthoimage 4 for mapping invasions patterns of *Pinus radiata* (EPSG: 32719)

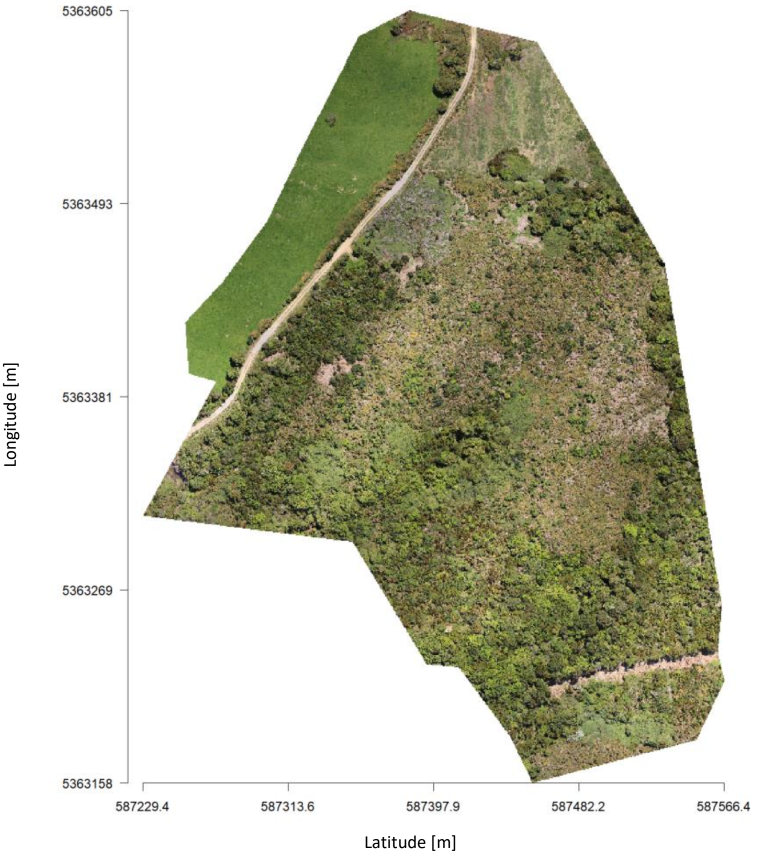

Fig S3-6: Orthoimage 1 for mapping invasions patterns of *Ulex europaeus* (EPSG: 32718)

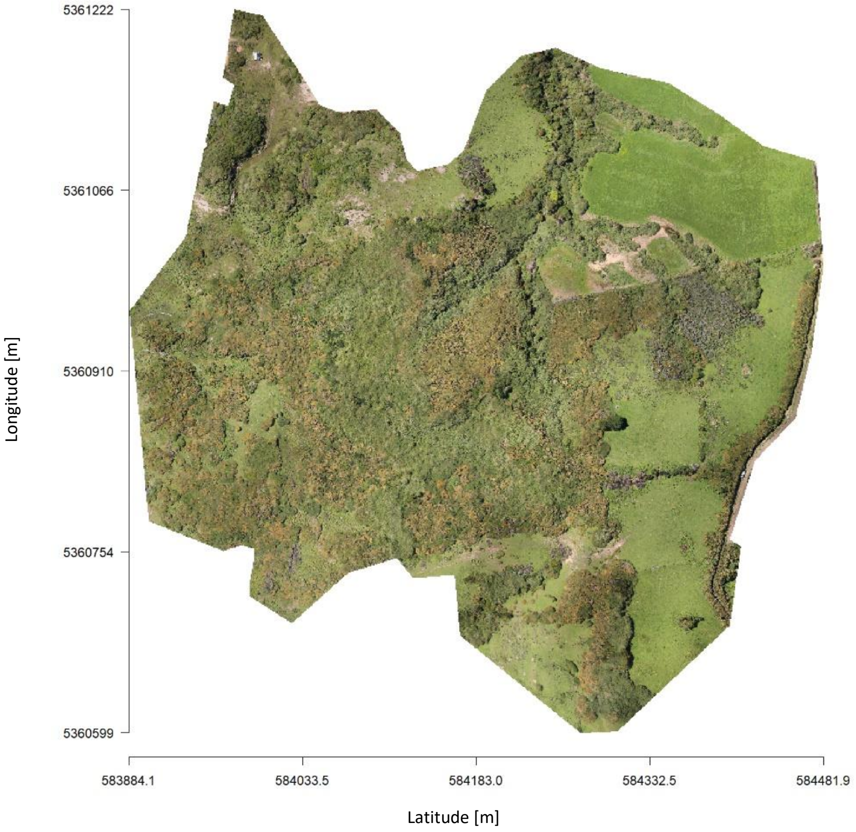

Fig S3-7: Orthoimage 2 for mapping invasions patterns of *Ulex europaeus* (EPSG: 32718)

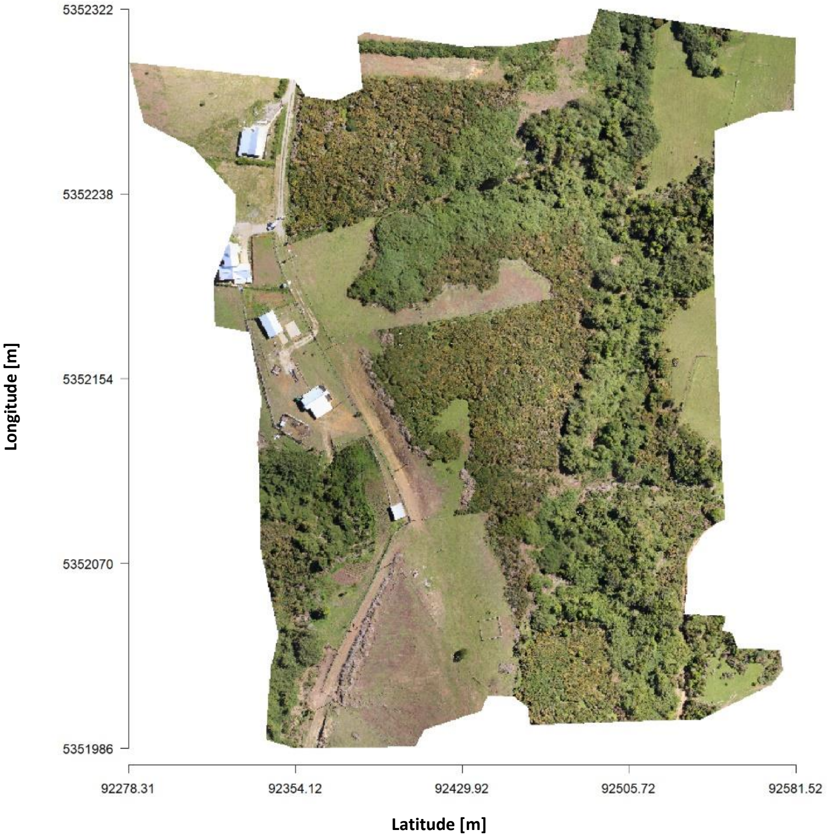

Fig S3-8: Orthoimage 3 for mapping invasions patterns of *Ulex europaeus* (EPSG: 32718)

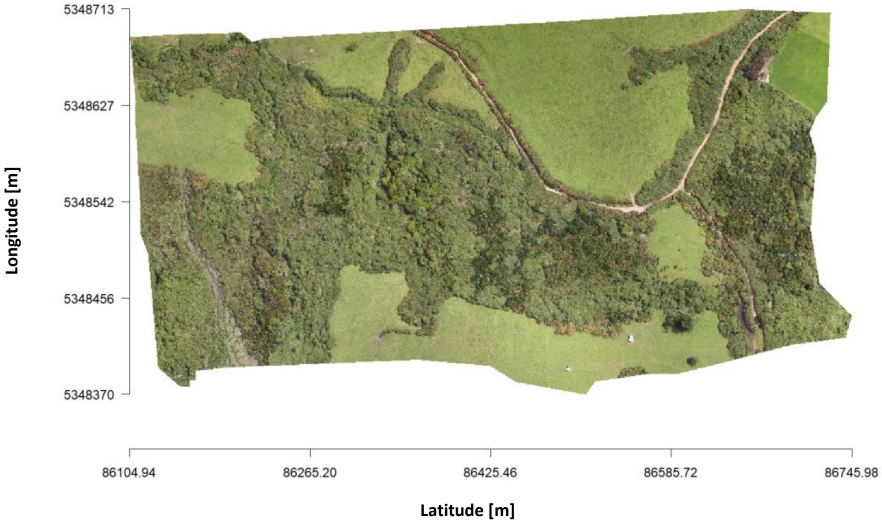

Fig S3-9: Orthoimage 4 for mapping invasions patterns of *Ulex europaeus* (EPSG: 32718)

the tree species *Metrosideros umbellata* and *Dacrydium cupressinum* (EPSG: 32758)

Supplementary information 2: Accuracy of the segmentation for all herbaceous vegetation communities found in the Mueller Glacier forefield.

Table S2-1: Predictive performance of segmenting the target classes using the Unet architecture

Target class	Accuracy [%]	Bias
Pioneer2	90	0.0529
Latesucc	92	0.0032
Intermed11	85	0.0720
Intermed12	86	0.0267
Intermed21	87	0.0045
Intermed22	89	0.0401
